# F254. EXPANDING THE REACH OF NAVIGATE CSC PROGRAMS ACROSS THE U.S.: WHAT DO WE KNOW?

**DOI:** 10.1093/schbul/sby017.785

**Published:** 2018-04-01

**Authors:** Piper Meyer-Kalos, Shaunequa James, Jillian Wright-Martin, Susan Gingerich, Shirley Glynn

**Affiliations:** 1 The University of Minnesota; 2 Independent Consultant; 3 UCLA

## Abstract

**Background:**

The Recovery After Initial Schizophrenia Episode-Early Treatment Program (RAISE-ETP) study was a landmark investigation whose positive results led to increased funding and support to build first episode psychosis programs across the US. Every state in the country received dedicated funding to implement a coordinated specialty care (CSC) program designed to identify and treat persons with first episode psychosis within the context of the nation’s multi-payer health system. Since the funding began in 2014, numerous CSC programs have been developed but little is known about which models of treatment providers are implementing and the success of these programs. The research here presents data from a survey focusing on providing feedback from the first episode psychosis programs in the US implementing NAVIGATE, the CSC program utilized in RAISE-ETP. The survey targets the program directors in the NAVIGATE programs; the aims of the survey include 1) to describe the program characteristics of NAVIGATE teams in the US and 2) to better understand how NAVIGATE programs are identifying and enrolling people into their services. Capturing local data on CSC team composition and case identification strategies is particularly critical in multi-payer systems lacking guidance and oversight from a national health system.

**Methods:**

An online survey is being conducted to assess the implementation of NAVIGATE programs in the US and evaluate the procedures that the program director utilizes to identify and enroll NAVIGATE participants in services. Program directors from NAVIGATE programs are being identified and contacted to participate by national trainers to join a national database of first episode programs. Program data collected includes information about the location of the program, staff in the different NAVIGATE team roles (prescriber, individual clinician, family clinician, and employment/education specialists, as well as optional roles such as peer advocate and case manager), program enrollment criteria, number of participants screened and enrolled, and rates of planned and unplanned discharge. In addition, program directors are asked questions to report community based strategies to identify participants and screening procedures to enroll participants. Data analysis will focus on presenting the demographic and clinical characteristics of the programs. Common themes will be ascertained, including barriers and facilitators to identifying and enrolling participants with first episode psychosis. Helpful recommendations provided by the project directors on identifying and screening participants will be synthesized and reported.

**Results:**

There are approximately 30 NAVIGATE programs in 14 states in the US. Results will highlight the dissemination of NAVIGATE in the US and implementation of these programs across a wide range of different communities. We will describe the dissemination of NAVIGATE across the US and similarities and differences across NAVIGATE programs. Results also will provide feedback on the challenges and helpful strategies that program directors have used to engage people in treatment.

**Discussion:**

The findings from this survey will be the first to provide an overview of the implementation of the NAVIGATE program in the US. The results will provide an overview of the dissemination of the NAVIGATE program, the only CSC program evaluated in a national US trial. Recommendations could help inform the ongoing development and dissemination of coordinated specialty care programs.

